# Trends in use of and complications from intrauterine contraceptive devices and tubal ligation or occlusion

**DOI:** 10.1186/s12978-017-0334-1

**Published:** 2017-06-08

**Authors:** Brandon Howard, ElizaBeth Grubb, Maureen J. Lage, Boxiong Tang

**Affiliations:** 1 Teva Global Medical Affairs, 41 Moores Road, Frazer, PA 19355 USA; 2 Teva Global Health Economics & Outcomes Research, 11100 Nall Ave, Overland Park, KS 66211 USA; 3 HealthMetrics Outcomes Research, 27576 River Reach Dr., Bonita Springs, FL 34134 USA; 4 Teva Global Health Economics & Outcomes Research, 41 Moores Road, Frazer, PA 19355 USA

**Keywords:** Copper IUD, Levonorgestrel IUD, Tubal sterilization

## Abstract

**Background:**

Long-acting reversible contraceptives such as intrauterine devices (IUDs) are highly effective in preventing pregnancy, cost effective, and increasing in popularity. It is unclear whether changes in IUD use are associated with changes in rates of irreversible tubal sterilization. In this analysis, we evaluate changes in rates of tubal sterilization, insertion of copper or levonorgestrel (LNG) IUDs, and related complications over time.

**Methods:**

Data were obtained from a retrospective claims database (Optum^TM^ Clinformatics^TM^ Data Mart) of women aged 15 to 45 years who underwent insertion of copper or LNG IUD or tubal sterilization between 1/1/2006 and 12/31/2011. Outcomes of interest included annual rates of insertion or sterilization and annual rates of potential complications and side effects.

**Results:**

The number of women included in the analysis each year ranged from 1,870,675 to 2,016,916. Between 2006 and 2011, copper IUD insertion claim rates increased from 0.18 to 0.25% and LNG IUD insertion claim rates increased from 0.63 to 1.15%, while sterilization claims decreased from 0.78 to 0.66% (*P* < 0.0001 for all comparisons). Increases in IUD insertion were apparent in all age groups; decreases in tubal sterilization occurred in women aged 20 to 34 years. The most common side effects and complications were amenorrhea (7.36–11.59%), heavy menstrual bleeding (4.85–15.69%), and pelvic pain (11.12–14.27%). Significant increases in claims of certain complications associated with IUD insertion or sterilization were also observed.

**Conclusion:**

Between 2006 and 2011, a decrease in sterilization rates accompanied an increase in IUD insertion rates, suggesting that increasing numbers of women opted for reversible methods of long-term contraception over permanent sterilization.

## Plain English summary

Long-acting reversible contraceptives such as intrauterine devices (IUDs) are among the most effective options for preventing pregnancy, and their popularity is increasing. However, whether changes in IUD use are associated with changes in rates of tubal sterilization, a largely irreversible and permanent contraceptive option, is unclear. In this study, we evaluated changes in rates of tubal sterilization, insertion of two different types of IUDs (copper or levonorgestrel [LNG] IUDs) and side effects associated with these devices over time. Data were obtained from an insurance claims database that included women aged 15 to 45 years who underwent insertion of copper or LNG IUD insertion or tubal sterilization between 1/1/2006 and 12/31/2011. Approximately 2 million women were included in the database each year. Between 2006 and 2011, copper IUD insertion rates increased from 0.18 to 0.25% and LNG IUD insertion rates increased from 0.63 to 1.15%, while sterilization claims decreased from 0.78 to 0.66%. Increases in IUD insertion were apparent in all age groups; decreases in tubal sterilization occurred in women aged 20 to 34 years. Results from our study suggest that increasing numbers of women are opting for copper and LNG IUDs over permanent sterilization.

## Background

Intrauterine devices (IUDs) are the most common method of reversible contraception [1, 2], used by approximately 14.3% of reproductive-aged women worldwide [3]. However, they are used only by 6.4% of American women using contraception [4]. Two commonly used IUDs in the US include the copper T380A IUD (copper IUD) and the levonorgestrel 20-mcg-releasing intrauterine device (LNG IUD). Both IUDs have been shown to be cost effective, have few contraindications, and are well tolerated [5–9].

Although the use of both IUDs is low in the US compared with the rest of the world, data suggest that use has substantially increased in recent years [10, 11]. Factors that may affect IUD use, including changes in rates of tubal sterilization, complications, or side effects have not been investigated.

In this report, we compare the use and complications associated with the copper IUD, LNG IUD, and tubal sterilization using data obtained from the Optum™ Clinformatics™ Data Mart database.

## Methods

The Optum™ Clinformatics™ Data Mart database is a large database of medical claims, pharmacy claims, lab results, and administrative data that contains information on patient characteristics, inpatient and outpatient encounters, and outpatient prescription drug coverage throughout the US. The database includes approximately 13 million unique individuals each year. Most individuals included in the database are commercially insured. The database is fully compliant with the Health Insurance Portability and Accountability Act.

This study evaluated claims from January 1, 2006, to December 31, 2011, among women aged 15–45 years as of the index date, defined as the insertion date of copper IUD (Healthcare Common Procedure Coding System [HCPCS] code J7300) or LNG IUD (HCPCS code J7302), or date of sterilization by tubal ligation/tubal occlusion, based on receipt of International Classification of Diseases, Ninth Revision, Clinical Modification (ICD-9-CM) codes 66.2 or 66.3 or Current Procedure Terminology codes 58600, 58605, 58611, 58615, 56870, or 56871.

Outcomes of interest included the rate of copper IUD insertion, LNG IUD insertion, and tubal sterilization by year (2006–2011) and by age group (ages 15–19, 20–24, 25–34, and 35–45), and changes in rate of potential complications and side effects over time. Complications and side effects were assessed based on receipt of ICD-9-CM codes for the following conditions: uterine perforation, pelvic inflammatory disease, post-insertion infection, dysmenorrhea, heavy menstrual bleeding (HMB), menorrhagia, anemia, ovarian cyst, pelvic pain, and amenorrhea.

All analyses were conducted using SAS®, version 9.3 (SAS Institute Inc., Cary, NC, USA). Chi square analyses were used to analyze categorical variables; analyses of variance were used to evaluate continuous variables. Findings with associated *P* values <0.05 were considered statistically significant.

## Results

**Fig. 1:**
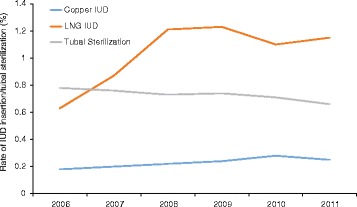
Rate of claims related to copper IUD insertion, LNG IUD insertion, and tubal sterilization by year of insertion/sterilization

The number of women included in the analysis each year ranged from 1,870,675 to 2,016,916. Rates of tubal sterilization decreased and rates of insertion of both copper and LNG IUDs increased between 2006 and 2011 (Table 1, Fig. 1). The percentage of women who underwent tubal ligation/tubal occlusion decreased from 0.78% (14,887/1,907,748) in 2006 to 0.66% (12,560/1,909,316) in 2011 (*P* < 0.0001), while rates of copper IUD insertion increased from 0.18% (3,454/1,907,748) to 0.25% (4,682/1,909,316) (*P* < 0.0001) and rates of LNG IUD insertion increased from 0.63% (12,028/1,907,748) to 1.15% (22,035/1,909,316) (*P* < 0.0001) from 2006 to 2011, respectively.

**Table 1:** Rate of IUD insertion and tubal sterilization over time

		Copper IUD	LNG IUD	Tubal Sterilization
Year	Total N	n (%)	n (%)	n (%)
2006	1,907,748	3454 (0.18)	12,028 (0.63)	14,887 (0.78)
Age 15 to 19 y	293,354	52 (0.02)	216 (0.07)	38 (0.01)
Age 20 to 24 y	220,950	330 (0.15)	1257 (0.57)	451 (0.20)
Age 25 to 34 y	566,152	1735 (0.31)	6230 (1.10)	6642 (1.17)
Age 35 to 45 y	827,292	1337 (0.16)	4325 (0.52)	7756 (0.94)
2007	1,940,301	3803 (0.20)	16,789 (0.87)	14,769 (0.76)
Age 15 to 19 y	299,599	79 (0.03)	416 (0.14)	42 (0.01)
Age 20 to 24 y	224,324	403 (0.18)	1813 (0.81)	444 (0.20)
Age 25 to 34 y	583,955	1964 (0.34)	8533 (1.46)	6471 (1.11)
Age 35 to 45 y	832,423	1357 (0.16)	6027 (0.72)	7812 (0.94)
2008	2,001,739	4474 (0.22)	24,276 (1.21)	14,667 (0.73)
Age 15 to 19 y	308,311	102 (0.03)	690 (0.22)	21 (0.01)
Age 20 to 24 y	237,192	455 (0.19)	2845 (1.2)	410 (0.17)
Age 25 to 34 y	615,537	2368 (0.38)	12,545 (2.04)	6473 (1.05)
Age 35 to 45 y	840,699	1549 (0.18)	8196 (0.97)	7763 (0.92)
2009	2,016,916	4868 (0.24)	24,811 (1.23)	14,881 (0.74)
Age 15 to 19 y	312,431	93 (0.03)	777 (0.25)	37 (0.01)
Age 20 to 24 y	237,723	460 (0.19)	2920 (1.23)	394 (0.17)
Age 25 to 34 y	625,323	2578 (0.41)	12,614 (2.02)	6232 (1.00)
Age 35 to 45 y	841,439	1737 (0.21)	8500 (1.01)	8218 (0.98)
2010	1,870,675	5246 (0.28)	20,639 (1.10)	13,313 (0.71)
Age 15 to 19 y	289,736	142 (0.05)	677 (0.23)	37 (0.010
Age 20 to 24 y	222,812	551 (0.25)	2101 (0.94)	243 (0.11)
Age 25 to 34 y	574,702	2782 (0.48)	10,316 (1.8)	5485 (0.95)
Age 35 to 45 y	783,425	1771 (0.23)	7545 (0.96)	7548 (0.96)
2011	1,909,316	4682 (0.25)	22,035 (1.15)	12,560 (0.66)
Age 15 to 19 y	295,377	116 (0.04)	762 (0.26)	24 (0.01)
Age 20 to 24 y	265,891	601 (0.23)	2648 (1.00)	280 (0.11)
Age 25 to 34 y	575,729	2451 (0.43)	10,694 (1.86)	5102 (0.89)
Age 35 to 45 y	772,319	1514 (0.20)	7931 (1.03)	7154 (0.93)
*P* Value for Trend Over Time in the Overall Population		<0.0001	<0.0001	<0.0001

**Fig. 2:**
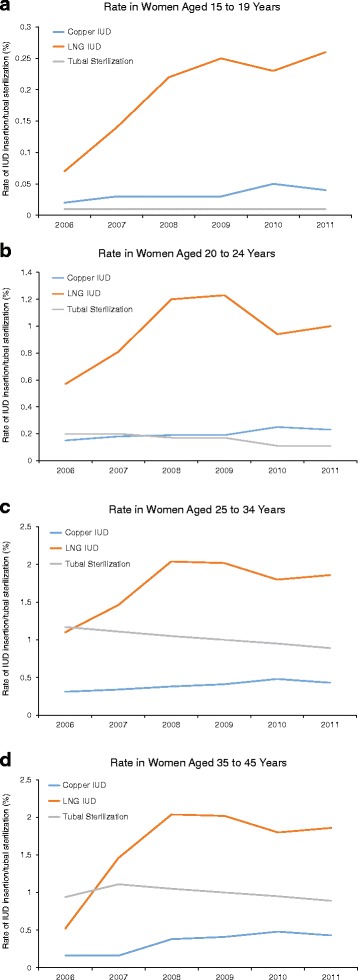
Rate of claims related to copper IUD insertion, LNG IUD insertion, and tubal sterilization by year of insertion or sterilization in women aged 15 to 19 years (**a**), 20 to 24 years (**b**), 25 to 34 years (**c**), and 35 to 45 years (**d**)

Increases in IUD insertion and decreases in tubal sterilization rates were apparent in most age groups (Table 1, Fig. 2). The greatest decreases in rates of sterilization occurred in women ages 25–34. Although insertion of either IUD in adolescents ages 15–19 was rare, the copper IUD insertion rate doubled and the LNG IUD insertion rate more than tripled in this age group between 2006 and 2011.

Rates of complications or side effects were low and are shown in Table 2. The most common side effects and complications were amenorrhea (7.36–11.59%), HMB (4.85–15.69%), and pelvic pain (11.12–14.27%). Significant increases over time were observed in rates of perforation of the uterine wall in all groups, HMB and menorrhagia with LNG IUD and tubal sterilization, dysmenorrhea and anemia with sterilization, and ovarian cysts with LNG IUD. A significant decrease in pelvic inflammatory disease was observed over time among women who underwent sterilization.

**Table 2:** Complications and side effects associated with IUD insertion and tubal sterilization over time

	Copper IUD	LNG IUD	Tubal Sterilization
Complications/Side Effect	n (%)	n (%)	n (%)
Amenorrhea (ICD-9 626.0)			
Total (2006–2011)	2385 (8.99)^ab^	9077 (7.53)^ac^	7050 (11.67)^bc^
2006	308 (8.92)	941 (7.82)	1638 (11.00)
2007	381 (10.02)	1278 (7.61)	1614 (10.93)
2008	412 (9.21)	1873 (7.72)	1700 (11.59)
2009	448 (9.20)	1845 (7.44)	1658 (11.14)
2010	452 (8.62)	1518 (7.36)	1429 (10.73)
2011	384 (8.20)	1622 (7.36)	1371 (10.92)
*P* Value	0.0775	0.4191	0.2768
Anemia (ICD-9 280.xx)			
Total (2006–2011)	595 (2.24)^b^	2832 (2.35)^c^	2003 (3.32)^bc^
2006	67 (1.94)	279 (2.32)	435 (2.92)
2007	78 (2.05)	386 (2.30)	433 (2.93)
2008	106 (2.37)	538 (2.22)	492 (3.35)
2009	119 (2.44)	600 (2.42)	513 (3.45)
2010	135 (2.57)	512 (2.48)	481 (3.61)
2011	90 (1.92)	517 (2.35)	469 (3.73)
*P* Value	0.1496	0.5243	0.0001
Dysmenorrhea (ICD-9 625.3)			
Total (2006–2011)	733 (2.76)^ab^	3909 (3.24)^ac^	2491 (4.12)^bc^
2006	90 (2.61)	378 (3.14)	587 (3.94)
2007	85 (2.50)	566 (3.37)	591 (4.00)
2008	132 (2.95)	723 (2.98)	598 (4.08)
2009	139 (2.86)	807 (3.25)	647 (4.35)
2010	137 (2.61)	692 (3.35)	649 (4.87)
2011	140 (2.99)	743 (3.37)	606 (4.82)
*P* Value	0.6388	0.1234	<0.0001
Heavy Menstrual Bleeding (ICD-9 626.2)			
Total (2006–2011)	1370 (5.16)^ab^	10 204 (8.46)^ac^	7328 (12.13)^bc^
2006	204 (5.91)	1048 (8.71)	1687 (11.33)
2007	198 (5.21)	1406 (8.37)	1750 (11.85)
2008	224 (5.01)	1846 (7.60)	1922 (13.10)
2009	256 (5.26)	1992 (8.03)	2045 (13.74)
2010	261 (4.98)	1881 (9.11)	2006 (15.07)
2011	227 (4.85)	2031 (9.22)	1971 (15.69)
*P* Value	0.3527	<0.0001	<0.0001
Infection (ICD-9 998.5x)			
Total (2006–2011)	15 (0.06)	88 (0.07)^c^	18 (0.03)^c^
2006	4 (0.12)	12 (0.10)	2 (0.01)
2007	1(0.03)	14 (0.08)	2 (0.01)
2008	6 (0.13)	15 (0.06)	7 (0.05)
2009	2 (0.04)	25 (0.10)	4 (0.03)
2010	0 (0.00)	14 (0.07)	4 (0.03)
2011	2 (0.04)	8 (0.04)	5 (0.04)
*P* Value	0.0543	0.1255	0.4301
Menorrhagia (ICD-9 627.0)			
Total (2006–2011)	53 (0.20)^ab^	528 (0.44)^ac^	410 (0.68)^bc^
2006	12 (0.35)	48 (0.40)	88 (0.59)
2007	6 (0.16)	58 (0.35)	94 (0.64)
2008	11 (0.25)	84 (0.35)	92 (0.63)
2009	12 (0.25)	89 (0.36)	119 (0.80)
2010	8 (0.15)	121 (0.59)	121 (0.91)
2011	4 (0.09)	128 (0.58)	126 (1.00)
*P* Value	0.1181	<0.0001	<0.0001
Ovarian Cyst (ICD-9 620.2)			
Total (2006–2011)	1157 (4.36)^ab^	6340 (5.26)^ac^	4324 (7.16)^bc^
2006	140 (4.05)	539 (4.48)	1045 (7.02)
2007	155 (4.08)	786 (4.68)	990 (6.70)
2008	211 (4.72)	1268 (5.22)	1076 (7.34)
2009	209 (4.29)	1400 (5.64)	1039 (6.98)
2010	223 (4.25)	1142 (5.53)	1003 (7.53)
2011	219 (4.68)	1205 (5.47)	910 (7.25)
*P* Value	0.5197	<0.0001	0.0975
Pelvic Inflammatory Disease (ICD-9 614.xx–616.xx)			
Total (2006–2011)	5053 (19.05)^ab^	19 063 (15.81)^ac^	11 162 (18.48)^bc^
2006	13 (0.38)	40 (0.33)	64 (0.43)
2007	12 (0.32)	53 (0.32)	66 (0.45)
2008	23 (0.51)	74 (0.30)	69 (0.47)
2009	15 (0.31)	60 (0.24)	54 (0.37)
2010	12 (0.23)	49 (0.24)	41 (0.31)
2011	18 (0.38)	58 (0.26)	25 (0.20)
*P* Value	0.282	0.3727	0.02
Pelvic Pain (ICD-9 625.9, 789.00)			
Total (2006–2011)	3222 (12.15)^ab^	13 891 (11.52)^ac^	8323 (13.78)^bc^
2006	384 (11.12)	1395 (11.60)	1987 (13.35)
2007	466 (12.25)	1915 (11.41)	1997 (13.52)
2008	519 (11.60)	2729 (11.24)	1976 (13.47)
2009	627 (12.88)	2897 (11.68)	2117 (14.23)
2010	661 (12.60)	2419 (11.72)	1881 (14.13)
2011	565 (12.07)	2536 (11.51)	1792 (14.27)
*P* Value	0.1449	0.617	0.0593
Perforation of Uterine Wall (ICD-9 621.8, 665.3)			
Total (2006–2011)	412 (1.55)^ab^	1558 (1.29)^ac^	387 (0.64)^bc^
2006	40 (1.16)	142 (1.18)	57 (0.38)
2007	51 (1.34)	176 (1.05)	76 (0.51)
2008	48 (1.07)	301 (1.24)	91 (0.62)
2009	88 (1.81)	319 (1.29)	119 (0.80)
2010	98 (1.87)	307 (1.49)	103 (0.77)
2011	87 (1.86)	313 (1.42)	109 (0.87)
*P* Value	0.0014	0.0023	<0.0001

^abc^Chi square pairwise comparisons between groups with the same superscript, *P* < 0.05. The pairwise comparisons were done for the total for each complication/side effect across years 2006–2011 and not for the individual years

## Discussion

Results indicate that tubal sterilization rates decreased and IUD insertion rates increased between 2006 and 2011. These findings were noted across all age groups, with the exception of sterilization in women ages 35–45, the rates of which were constant. By 2008, insertion rates of LNG IUD exceeded rates of sterilization in every age group, including women ages 35–45. Importantly, substantial increases in insertion rates for both the copper and LNG IUDs were seen in younger women, including adolescents.

Results suggesting an increase in IUD use are consistent with data from the National Survey of Family Growth, which showed that from 2002 to 2013, the prevalence of IUD use increased from 2.0 to 10.3% among female contraceptive users aged 15–44 years [12]. The prevalence of female sterilization in the same population decreased from 27.0 to 25.1% over the same time period. In a separate analysis of sexually active women aged 15–24 years, IUD use increased from 0.2 to 2.5% in teens ages 15–19 and from 2.0 to 5.4% in women aged 20–24 years, although the increase was primarily observed in parous women [11]. Another retrospective cohort study found that IUD insertion rates increased nearly 7-fold between 2002 and 2009 [6].

Importantly, women experienced few complications with either IUD. Differences in complication rates between IUDs were of minimal clinical significance. The most frequent complications in both IUD groups were menstrual disorders and pelvic pain; however, patients who underwent tubal sterilization reported these adverse effects more frequently than IUD users. The most serious complications associated with IUD use, such as uterine perforation and pelvic inflammatory disease, were reported in fewer than 2% of women.

Limitations of the study included its observational, retrospective nature, lack of representation of women without health insurance, and nature of claims databases. Despite these limitations, our findings confirm recent data suggesting a shift toward long-acting reversible contraceptive methods and away from permanent methods. Copper IUD and LNG IUD insertion and tubal sterilization were associated with a low rate of complications.

## Conclusions

Our analysis of a retrospective claims database supported an increase in women selecting reversible methods of long-term contraception over permanent tubal sterilization, as shown by an increase in copper IUD and LNG IUD insertion rates and decreased sterilization rates between 2006 and 2011. Younger women showed substantial increases in IUD insertion rates. Among all women, rates of complications or side effects were low.

## Abbreviations

HCPCS: Healthcare Common Procedure Coding System
HMB: Heavy menstrual bleeding
ICD-9-CM: International Classification of Diseases, Ninth Revision, Clinical Modification
IUD: Intrauterine device
LNG: Levonorgestrel
