# Correction to: Trends in use of and complications from intrauterine contraceptive devices and tubal ligation or occlusion

**DOI:** 10.1186/s12978-017-0386-2

**Published:** 2017-10-06

**Authors:** Brandon Howard, ElizaBeth Grubb, Maureen J. Lage, Boxiong Tang

**Affiliations:** 1 Teva Global Medical Affairs, 41 Moores Road, Frazer, PA 19355 USA; 2 Teva Global Health Economics & Outcomes Research, 11100 Nall Ave, Overland Park, KS 66211 USA; 3 HealthMetrics Outcomes Research, 27576 River Reach Dr., Bonita Springs, FL 34134 USA; 4 Teva Global Health Economics & Outcomes Research, 41 Moores Road, Frazer, PA 19355 USA

As result of a query from a reader, the authors reviewed the data in Table 2 of the original article [1] and found a number of errors in the prior construction of this data table. A formatting error has also been noticed in Table 1.

**Table 1:** Rate of IUD insertion and tubal sterilization over time

		Copper IUD	LNG IUD	Tubal Sterilization
Year	Total N	n (%)	n (%)	n (%)
2006	1,907,748	3454 (0.18)	12,028 (0.63)	14,887 (0.78)
Age 15 to 19 y	293,354	52 (0.02)	216 (0.07)	38 (0.01)
Age 20 to 24 y	220,950	330 (0.15)	1257 (0.57)	451 (0.20)
Age 25 to 34 y	566,152	1735 (0.31)	6230 (1.10)	6642 (1.17)
Age 35 to 45 y	827,292	1337 (0.16)	4325 (0.52)	7756 (0.94)
2007	1,940,301	3803 (0.20)	16,789 (0.87)	14,769 (0.76)
Age 15 to 19 y	299,599	79 (0.03)	416 (0.14)	42 (0.01)
Age 20 to 24 y	224,324	403 (0.18)	1813 (0.81)	444 (0.20)
Age 25 to 34 y	583,955	1964 (0.34)	8533 (1.46)	6471 (1.11)
Age 35 to 45 y	832,423	1357 (0.16)	6027 (0.72)	7812 (0.94)
2008	2,001,739	4474 (0.22)	24,276 (1.21)	14,667 (0.73)
Age 15 to 19 y	308,311	102 (0.03)	690 (0.22)	21 (0.01)
Age 20 to 24 y	237,192	455 (0.19)	2845 (1.2)	410 (0.17)
Age 25 to 34 y	615,537	2368 (0.38)	12,545 (2.04)	6473 (1.05)
Age 35 to 45 y	840,699	1549 (0.18)	8196 (0.97)	7763 (0.92)
2009	2,016,916	4868 (0.24)	24,811 (1.23)	14,881 (0.74)
Age 15 to 19 y	312,431	93 (0.03)	777 (0.25)	37 (0.01)
Age 20 to 24 y	237,723	460 (0.19)	2920 (1.23)	394 (0.17)
Age 25 to 34 y	625,323	2578 (0.41)	12,614 (2.02)	6232 (1.00)
Age 35 to 45 y	841,439	1737 (0.21)	8500 (1.01)	8218 (0.98)
2010	1,870,675	5246 (0.28)	20,639 (1.10)	13,313 (0.71)
Age 15 to 19 y	289,736	142 (0.05)	677 (0.23)	37 (0.01)
Age 20 to 24 y	222,812	551 (0.25)	2101 (0.94)	243 (0.11)
Age 25 to 34 y	574,702	2782 (0.48)	10,316 (1.8)	5485 (0.95)
Age 35 to 45 y	783,425	1771 (0.23)	7545 (0.96)	7548 (0.96)
2011	1,909,316	4682 (0.25)	22,035 (1.15)	12,560 (0.66)
Age 15 to 19 y	295,377	116 (0.04)	762 (0.26)	24 (0.01)
Age 20 to 24 y	265,891	601 (0.23)	2648 (1.00)	280 (0.11)
Age 25 to 34 y	575,729	2451 (0.43)	10,694 (1.86)	5102 (0.89)
Age 35 to 45 y	772,319	1514 (0.20)	7931 (1.03)	7154 (0.93)
*P* Value for Trend Over Time in the Overall Population		<0.0001	<0.0001	<0.0001

The correct version of Table 1 and Table 2 is published in this erratum.

**Table 2:** Complications and side effects associated with IUD insertion and tubal sterilization over time

	Copper IUD	LNG IUD	Tubal Sterilization
Complications/Side Effect	n (%)	n (%)	n (%)
Amenorrhea (ICD-9 626.0)			
Total (2006–2011)	2385 (8.99)^ab^	9077 (7.53)^ac^	9410 (11.06)^bc^
2006	308 (8.92)	941 (7.82)	1638 (11.00)
2007	381 (10.02)	1278 (7.61)	1614 (10.93)
2008	412 (9.21)	1873 (7.72)	1700 (11.59)
2009	448 (9.20)	1845 (7.44)	1658 (11.14)
2010	452 (8.62)	1518 (7.36)	1429 (10.73)
2011	384 (8.20)	1622 (7.36)	1371 (10.92)
* P* Value	0.0775	0.4191	0.2768
Anemia (ICD-9 280.xx)			
Total (2006–2011)	595 (2.24)^b^	2832 (2.35)^c^	2823 (3.32)^bc^
2006	67 (1.94)	279 (2.32)	435 (2.92)
2007	78 (2.05)	386 (2.30)	433 (2.93)
2008	106 (2.37)	538 (2.22)	492 (3.35)
2009	119 (2.44)	600 (2.42)	513 (3.45)
2010	135 (2.57)	512 (2.48)	481 (3.61)
2011	90 (1.92)	517 (2.35)	469 (3.73)
* P* Value	0.1496	0.5243	0.0001
Dysmenorrhea (ICD-9 625.3)			
Total (2006–2011)	723 (2.73)^ab^	3909 (3.24)^ac^	3678 (4.32)^bc^
2006	90 (2.61)	378 (3.14)	587 (3.94)
2007	85 (2.24)	566 (3.37)	591 (4.00)
2008	132 (2.95)	723 (2.98)	598 (4.08)
2009	139 (2.86)	807 (3.25)	647 (4.35)
2010	137 (2.61)	692 (3.35)	649 (4.87)
2011	140 (2.99)	743 (3.37)	606 (4.82)
* P* Value	0.6388	0.1234	<0.0001
Heavy Menstrual Bleeding (ICD-9 626.2)			
Total (2006–2011)	1370 (5.16)^ab^	10,204 (8.46)^ac^	11,381 (13.38)^bc^
2006	204 (5.91)	1048 (8.71)	1687 (11.33)
2007	198 (5.21)	1406 (8.37)	1750 (11.85)
2008	224 (5.01)	1846 (7.60)	1922 (13.10)
2009	256 (5.26)	1992 (8.03)	2045 (13.74)
2010	261 (4.98)	1881 (9.11)	2006 (15.07)
2011	227 (4.85)	2031 (9.22)	1971 (15.69)
* P* Value	0.3527	<0.0001	<0.0001
Infection (ICD-9 998.5×)			
Total (2006–2011)	15 (0.06)	88 (0.07)^c^	24 (0.03)^c^
2006	4 (0.12)	12 (0.10)	2 (0.01)
2007	1 (0.03)	14 (0.08)	2 (0.01)
2008	6 (0.13)	15 (0.06)	7 (0.05)
2009	2 (0.04)	25 (0.10)	4 (0.03)
2010	0 (0.00)	14 (0.07)	4 (0.03)
2011	2 (0.04)	8 (0.04)	5 (0.04)
* P* Value	0.0543	0.1255	0.4301
Menorrhagia (ICD-9 627.0)			
Total (2006–2011)	53 (0.20)^ab^	528 (0.44)^ac^	640 (0.75)^bc^
2006	12 (0.35)	48 (0.40)	88 (0.59)
2007	6 (0.16)	58 (0.35)	94 (0.64)
2008	11 (0.25)	84 (0.35)	92 (0.63)
2009	12 (0.25)	89 (0.36)	119 (0.80)
2010	8 (0.15)	121 (0.59)	121 (0.91)
2011	4 (0.09)	128 (0.58)	126 (1.00)
* P* Value	0.1181	<0.0001	<0.0001
Ovarian Cyst (ICD-9 620.2)			
Total (2006–2011)	1157 (4.36)^ab^	6340 (5.26)^ac^	6063 (7.13)^bc^
2006	140 (4.05)	539 (4.48)	1045 (7.02)
2007	155 (4.08)	786 (4.68)	990 (6.70)
2008	211 (4.72)	1268 (5.22)	1076 (7.34)
2009	209 (4.29)	1400 (5.64)	1039 (6.98)
2010	223 (4.25)	1142 (5.53)	1003 (7.53)
2011	219 (4.68)	1205 (5.47)	910 (7.25)
* P* Value	0.5197	<0.0001	0.0975
Pelvic Inflammatory Disease (ICD-9 614.xx-616.xx)			
Total (2006–2011)	93 (0.35)	334 (0.28)^c^	319 (0.37)^c^
2006	13 (0.38)	40 (0.33)	64 (0.43)
2007	12 (0.32)	53 (0.32)	66 (0.45)
2008	23 (0.51)	74 (0.30)	69 (0.47)
2009	15 (0.31)	60 (0.24)	54 (0.36)
2010	12 (0.23)	49 (0.24)	41 (0.31)
2011	18 (0.38)	58 (0.26)	25 (0.20)
* P* Value	0.282	0.3727	0.02
Pelvic Pain (ICD-9 625.9, 789.00)			
Total (2006–2011)	3222 (12.15)^ab^	13,891 (11.52)^ac^	11,750 (13.81)^bc^
2006	384 (11.12)	1395 (11.60)	1987 (13.35)
2007	466 (12.25)	1915 (11.41)	1997 (13.52)
2008	519 (11.60)	2729 (11.24)	1976 (13.47)
2009	627 (12.88)	2897 (11.68)	2117 (14.23)
2010	661 (12.60)	2419 (11.72)	1881 (14.13)
2011	565 (12.07)	2536 (11.51)	1792 (14.27)
* P* Value	0.1449	0.617	0.0593
Perforation of Uterine Wall (ICD-9 621.8, 665.3)			
Total (2006–2011)	412 (1.55)^ab^	1558 (1.29)^ac^	555 (0.65)^bc^
2006	40 (1.16)	142 (1.18)	57 (0.38)
2007	51 (1.34)	176 (1.05)	76 (0.51)
2008	48 (1.07)	301 (1.24)	91 (0.62)
2009	88 (1.81)	319 (1.29)	119 (0.80)
2010	98 (1.87)	307 (1.49)	103 (0.77)
2011	87 (1.86)	313 (1.42)	109 (0.87)
* P* Value	0.0014	0.0023	<0.0001

^abc^Chi square pairwise comparisons between groups with the same superscript, *p* < 0.05. The pairwise comparisons were done for the total for each complication/side effect across years 2006–2011 and not for the individual years
